# A Treatise on Diseases of the Eye

**Published:** 1847-07

**Authors:** 


Art. V.—*A Treatise on the Diseases of the Eye.* By W. Lawrence, F.R.S., Surgeon Extraordinary to the Queen, etc. etc. etc. *A new edition. Edited, with numerous Additions, and one hundred and seventy-six Illustrations,* by Isaac Hays, M.D., Surgeon to Wills's Hospital, Physician to the Philadelphia Orphan Asylum; Member of the American Philosophical Society; Fellow of the College of Physicians of Philadelphia, etc. etc. 8vo. pp. 859. Philadelphia: Lea and Blanchard: 1847.

This work ranks among the classics in surgery, and all that we propose is, to announce its reappearance in an enlarged and improved form. Mr. Lawrence stands in the foremost rank of English surgeons, and high among the foremost. As a writer, we are not sure that modern surgery has produced one equal to him in vigor and philosophical acuteness. In the work before us he shows himself the practical surgeon, familiar with his subject and with the wants of his readers. It commences with a full account of the anatomy and physiology of the eyeball, and to this succeeds a description of the various lesions of structure and function. After these preliminary chapters the author goes on with his history of the manifold diseases of the eye, pointing out the remedies appropriate to each. Dr. Hays has contributed many original facts to this edition, and thereby enhanced its value to the student and practitioner.

